# A method to experimentally clamp leaf water content to defined values to assess its effects on apoplastic pH

**DOI:** 10.1186/s13007-022-00905-y

**Published:** 2022-05-30

**Authors:** Hartmut Kaiser, Amit Sagervanshi, Karl H. Mühling

**Affiliations:** 1 Institut Für Pflanzenernährung and Bodenkunde, Christian-Albrechts-Universität zu Kiel, Hermann-Rodewald-Straße 2, 24098 Kiel, Germany; 2 Present Address: Botanisches Institut und Botanischer Garten der Christian-Albrechts-Universität zu Kiel, Am Botanischen Garten 7, 24098 Kiel, Germany

**Keywords:** Leaf water content, Air humidity, Transpiration, Apoplast, pH, Sensor, Feedback, Fluorescence ratio

## Abstract

**Background:**

Leaf hydration is controlled by feedback mechanisms, e.g. stomatal responses, adjustments of osmotic potential and hydraulic conductivity. Leaf water content thus is an input into related feedback-loops controlling the balance of water uptake and loss.

Apoplastic alkalisation upon leaf dehydration is hypothesized to be involved together and in interaction with abscisic acid (ABA) in water stress related signaling on tissue level. However, important questions are still unresolved, e.g. the mechanisms leading to pH changes and the exact nature of its interaction with ABA. When studying these mechanisms and their intermediate signaling steps, an experimenter has only poor means to actually control the central experimental variable, leaf water content (LWC), because it is not only dependent on external variables (e.g. air humidity), which are under experimental control, but is also governed by the biological influences controlling transpiration and water uptake. Those are often unknown in their magnitude, unpredictable and fluctuating throughout an experiment and will prevent true repetitions of an experiment. The goal of the method presented here is to experimentally control and manipulate leaf water content (LWC) of attached intact leaves enclosed in a cuvette while simultaneously measuring physiological parameters like, in this case, apoplastic pH.

**Results:**

An experimental setup was developed where LWC is measured by a sensor based on IR-transmission and its signal processed to control a pump which circulates air from the cuvette through a cold trap. Hereby a feedback-loop is formed, which by adjusting vapour pressure deficit (VPD) and consequently leaf transpiration can precisely control LWC. This technique is demonstrated here in a combination with microscopic fluorescence imaging of apoplastic pH (pH_apo_) as indicated by the excitation ratio of the pH sensitive dye OregonGreen. Initial results indicate that pH_apo_ of the adaxial epidermis of *Vicia faba* is linearly related to reductions in LWC.

**Conclusions:**

Using this setup, constant LWC levels, step changes or ramps can be experimentally applied while simultaneously measuring physiological responses. The example experiments demonstrate that bringing LWC under experimental control in this way allows better controlled and more repeatable experiments to probe quantitative relationships between LWC and signaling and regulatory processes.

## Introduction

Leaf water content (LWC) is one of the most important properties for biochemical functioning, growth, and plant survival. Therefore it is under tight physiological control by diverse mechanisms [16]. Regulation of LWC generally occurs via negative feedback loops. Deviations of LWC or related tissue properties like cell turgor are perceived and fed into signal transduction networks, finally resulting in physiological responses stabilizing an optimal LWC [17]. Among other mechanisms, osmotic adjustment and modulation of hydraulic conductance [19] serve to maintain a stable and beneficial level of leaf hydration [18]. However, the most effective control over LWC is exerted by the adjustment of stomatal apertures and thus transpiration, which can quickly change the leaf water balance [2, 3]. It is well established that stomatal guard cells respond to changing leaf hydration with osmotic adjustments, resulting in stomatal aperture changes. Whereas the signal transduction and membrane processes in guard cells are understood in depth [1, 9], the intercellular signaling of water stress to guard cells is still partly obscure. The prominent role of abscisic acid in plant water homeostasis is evident, but other factors emerge as important alternative signaling agents or factors which modulate ABA-signaling [13]. Apoplastic pH (pH_apo_) is hypothesized to play a role in signaling water stress within the tissue either directly or by changing the distribution of ABA between leaf compartments with different pH levels [4, 7, 8, 12]. Important questions, however, are up to now unresolved. Especially the mechanisms behind the change of pH_apo_ in response to variations in LWC and the transduction of pH_apo_ into cellular responses are not yet understood. Experiments targeting these questions are difficult because methods to experimentally manipulate leaf hydration in an exact and repeatable manner are lacking.

Research into the physiological and molecular feedback mechanisms of leaf water homeostasis usually involves an experimental treatment influencing leaf water status, measurements of intermediate physiological events and/or of the resulting effect on water flows and leaf hydration. In such experiments, the treatment should ideally and repeatably manipulate the property which serves as input into the feedback cycles. While it is not sure which one of the water-status related physicochemical properties are sensed in cells, be it e.g. turgor pressure, osmolarity or water potential, the absolute amount of water in the tissue is arguably a central property the experimenter should control precisely and repeatably. Unfortunately, direct control of LWC is hard to achieve. While it is easy to exactly define the external conditions which impact leaf water content like vapour pressure deficit, osmotic potential of solutions, soil water potential etc., the resulting LWC also depends on biological parameters, which are not under experimental control and can change during the experiment, like degree of stomatal opening. Therefore even if, e.g. VPD can be precisely and repeatably controlled, and be kept constant during the experimental period, the resulting LWC is subject to the varying balance of water uptake and transpirational flow dependent on stomatal responses, osmotic adjustment of cell sap and variations in hydraulic conductivity. Therefore, LWC will vary between repetitions and be subject to uncontrolled change during the experiment. In the absence of a method to measure LWC, it will not even be known.

Here we present an experimental approach to precisely control LWC by feeding the signal of a recently developed optical LWC sensor into the humidity control loop of a cuvette to dynamically adjust air humidity levels necessary to maintain the desired LWC. Concomitant measurements of apoplastic pH by ratiometric fluorescence imaging [6, 14] allow testing hypotheses regarding the role of apoplastic pH as a tissue signaling agent in leaf water homeostasis.

## Method

**Fig. 1:**
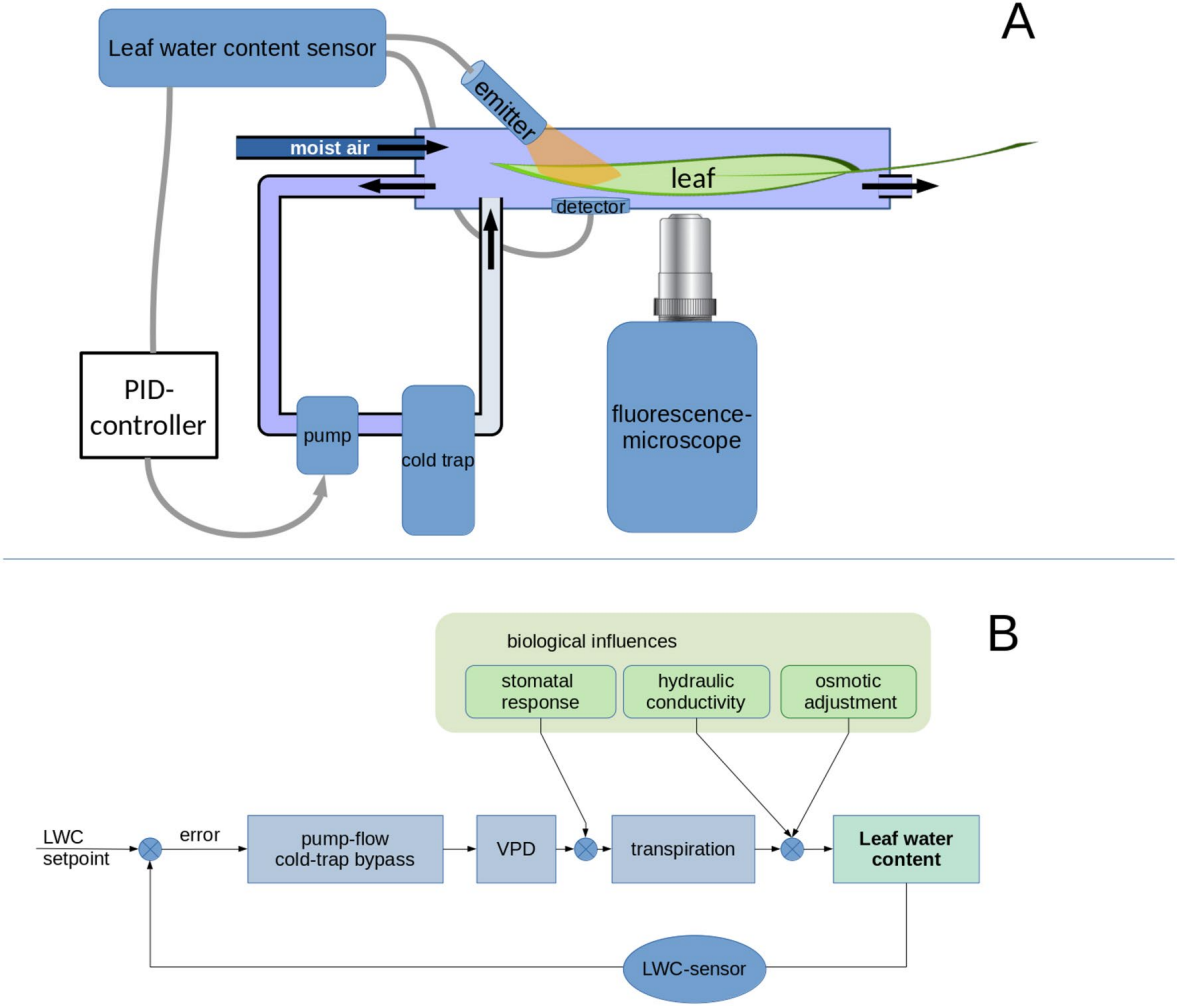
**A** Scheme of the experimental setup and feedback loop. **B** control diagram of the feedback loop controlling leaf water content (LWC) by using the LWC sensor's output to control the pump in the drying bypass loop. Biological regulators of leaf water homeostasis (green boxes) modify the effect of vapour pressure deficit (VPD) on LWC. Stomatal responses determine the effect of VPD on transpiration while hydraulic conductivity and osmotic adjustment modulate the effect of transpiration on the leaf water balance and thus LWC. The varying effect of these biological influences is compensated for by continuous adjustment of VPD

**Fig. 2:**
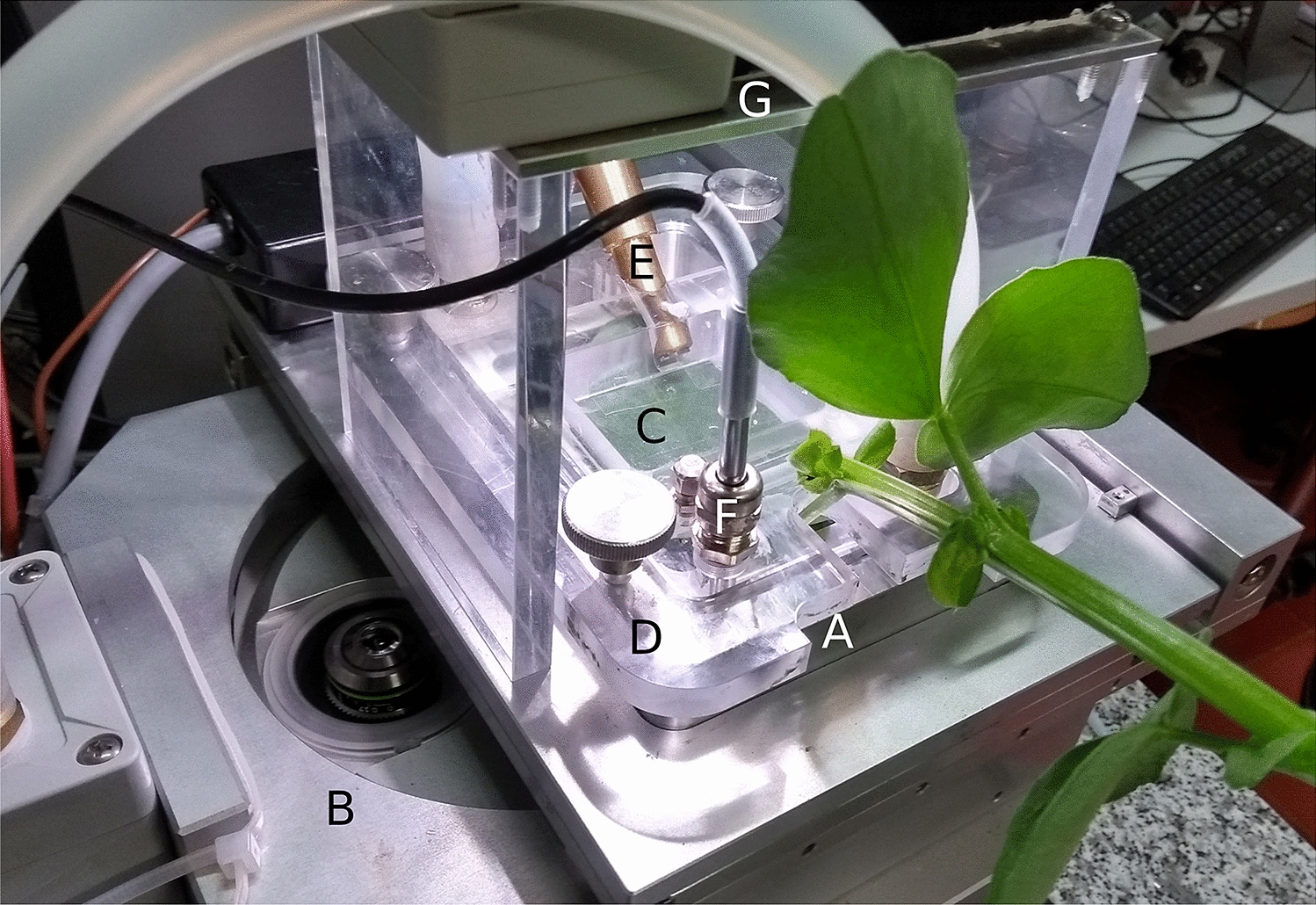
Cuvette for control of leaf water content of the enclosed leaf. The cuvette **A** is installed on the motorized stage of an inverted fluorescence microscope **B**. The enclosed leaf **C** is attached to the transparent **D** lid of the cuvette with double sided tape and microscopically observed through the glass bottom made of cover slide glass. A leaf water content sensor comprised of a dual IR-LED light source **E** and a photo-diode (not visible) below the leaf-surface continuously tracks LWC. Its output is fed into a feedback algorithm which controls vapour pressure deficit (VPD) of the air passing over the leaf surface. Alternatively VPD can be feed-back-controlled according to the output of a air humidity sensor **F**. Illumination is provided by a switchable white LED light source under the control of the microscope central unit, which can be switched off for about 120 ms during fluorescence measurements

Plants of *Vicia faba* var. Fuego (NPZ, Gettorf, Germany) used in these experiments were hydroponically grown in a controlled climatic chamber (light: PPFD 200 µmol m^−2^ s^−1^ 14/10 h day/night; temperature 20/15 °C; relative humidity 50/60% humidity) as decribed in more detail in [15]. Fully expanded attached leaves of c. 3 weeks old plants were mounted in a humidity-controlled cuvette (volume c. 110cm^3^) with forced flow across the lower leaf surface. The cuvette has a glass bottom (thickness 0.17 mm) and is mounted on top of a microscopic stage of an inverted microscope (iMIC, FEI Munich GmbH, now Thermo Fisher), allowing ratiometric fluorescence microscopy (Figs. 1, 2). Humidity control was achieved by streaming a constant flow (1–3 l * min^−1^)of air humidified to saturation in a washing bottle into the cuvette and removing varying amounts of humidity with a cold trap (KF-18/2, Walz, Germany) arranged in a bypass flow which was driven by a variable flow pump (LD10G, Walz) (Fig. 1). In this way a VPD between c. 0.25 and 2.5 could be achieved. A LWC sensor based on an IR transmission ratio in the near infrared range [10] continuously measured LWC near the pH measurement site (Fig. 2). In brief, this sensor measured the transmission through the leaf lamina of infrared light at two spectral bands around 1450 nm (which is sensitive to liquid water) and around 1050 nm which is less affected by liquid water and serves as a reference. LEDs at 1450 nm and 1050 nm directed to the upper leaf surface at an angle of 45 were used as lightsources and driven by pulsed (1.2 kHz) constant current sources with a current of about 20 mA. Transmitted measurement light was picked up by an InGaAs photodiode placed below the leaf. The signals were amplified by a transimpedance amplifier and fed into a lock-in amplifier for phase sensitive amplification, effectively excluding ambient light from the signal. The ratio of I1450/I1050 can be calibrated against gravimetrically obtained LWC. LWC and VPD were continuously recorded by a datalogger (CR23x, Campbell Scientific). A proportional, integral and derivative (PID) feedback loop [11] encoded into the same dataloggers program generated a variable voltage which controlled the bypass pump and thereby VPD in the cuvette. This feedback signal depended either on-air humitity measured by a relative humidity sensor (SHT31-A, Sensirion) in the cuvette or on deviations of measured LWC from experimental LWC setpoints. The PID algorithm followed the classical formulation with

$$\begin{array}{c}Output={K}_{p}\cdot e\left(t\right)+{K}_{i}\cdot \int e\left(t\right)dt+{K}_{d}\cdot \frac{d}{dt}\cdot e\left(t\right)\\ \\ \end{array}$$

where output = voltage fed into the bypass pump; e = error = setpoint – input; t= time; Kp = proportionality constant; Ki = integrative constant; Kd = differential constant

PID parameters were determined by trial and error to achieve rapid LWC adjustments with minimal overshoot. After tuning the feedback loop, the useful Kd turned out to be very small, therefore a PI-controller would probably also be able to control LWC.

In-situ measurements of pH apo were performed using the ratiometric pH-sensitive dye OregonGreen dextrane (Invitrogen D7170) [5]. This dye has its best sensitivity between pH 4.5 to 6.5 and is thus applicable in the acidic apoplast. Calibration was performed by measuring fluorescence ratio with the iMIC with the same optical settings as used during the experiments on a series of buffer solutions in the range from pH 3 to pH 7.5 in rectangle capillaries (Vitrotubes.com, # 5015-050). For pH measurements the OregonGreen solution (50 µM) was injected into the apoplast with a blunt syringe the afternoon before the measurement before installation into the cuvette to allow leaf acclimatization over night. Fluorescence images were acquired using a 50×objective (LMPLanFLN 50x/0.50 WD 10.5 mm) using excitation light at 440 and 490 nm provided by a Till Polychrome V Monochromator (Fei Munich GmbH) with a 150 W xenon arc burner and a filter cube for OregonGreen (F37-468/F37-533/F48-510). The fluorescence camera (Andor Clara E, 1344 × 1024px, cooled to − 12 °C) captured images with exposure times of usually 120 ms but sometimes adjusted as required to reach a suitable signal level.

## Results and discussion

**Fig. 3:**
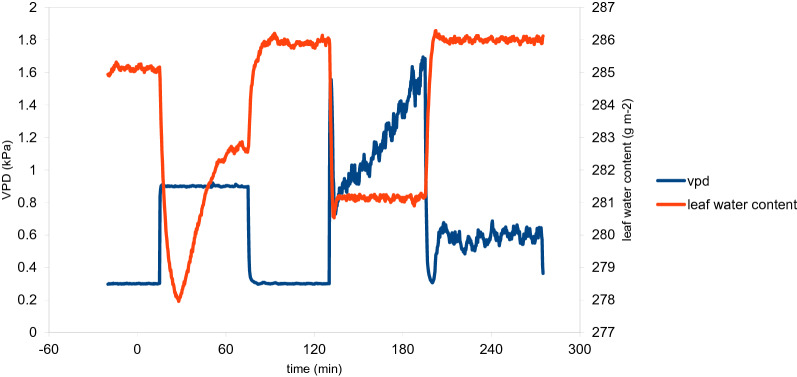
Measurement of leaf water content (LWC) and vapour pressure deficit (VPD) during an experiment using first (0–120 min) feedback-control of air humidity to perform a step change in air humidity and (120–280 min) feedback control of LWC to perform step changes of LWC. In the first part, leaf water content fluctuates dynamically as a result of changing leaf water balance. In the second part, LWC is fixed, and VPD is varied by feedback control as required to clamp LWC at constant set-points

The functioning of the experimental setup to control of LWC was demonstrated in a two-part experiment, contrasting conventional humidity control with feedback control of LWC (Fig. 3). First, a stepwise change in VPD from 0.3 to 0.9 for 1 h was performed, and the response of LWC observed. LWC responded to a sudden step-wise increase of VPD with an initial decline of LWC to a minimum at 10 min and a gradual recovery during the following 50 min, which, although not recorded in this experiment, can likely be attributed to stomatal closure. After 1 h LWC had substantially recovered under unchanged VPD. Upon return to low VPD, LWC returned to near saturation values within 10 min following a typical relaxation kinetic. Notably, although VPD was kept precisely constant during this part of the experiment, LWC displayed a complex dynamic response governed by perturbation and relaxation of the water balance and biological adjustment responses. For the experimenter it would be hard to predict the effect of VPD changes on LWC or to to carry out this treatment in a reproducible manner, as the effect of VPD on LWC depends on various unknowns, like the initial degree of stomatal opening and the stomatal response to this treatment.

This was the reason to program an alternative feedback loop to clamp LWC instead of VPD to values defined by the experimenter, by feeding the output of the LWC sensor into feedback control (Fig. 1). In this second part of the experiment shown in Fig. 3, feedback control of LWC was used to step-wise reduce LWC. The LWC changes were much faster and occurred within minutes lacking most of the slur observed under feed-back control of VPD. During the 1 h period, LWC was kept constant while VPD was adjusted in the way required to control LWC. This automatically compensated fo biological influences like changes of stomatal apertures (Fig. 1B). VPD in this experiment thus should no longer be seen as the independet experimental variable which should be kept as constant and repeatable as possible but as the means to achieve the desired LWC. The peaks and jitters observed in the VPD trace reflect the feedback loop's action necessary to stabilize LWC at the set-point. Strikingly, the compensatory action of the feedback loop is apparent in the gradual increase of VPD during the 1 h period, which was necessary to counteract the biological reactions of stomatal closure and possibly osmotic adjustment.

**Fig. 4:**
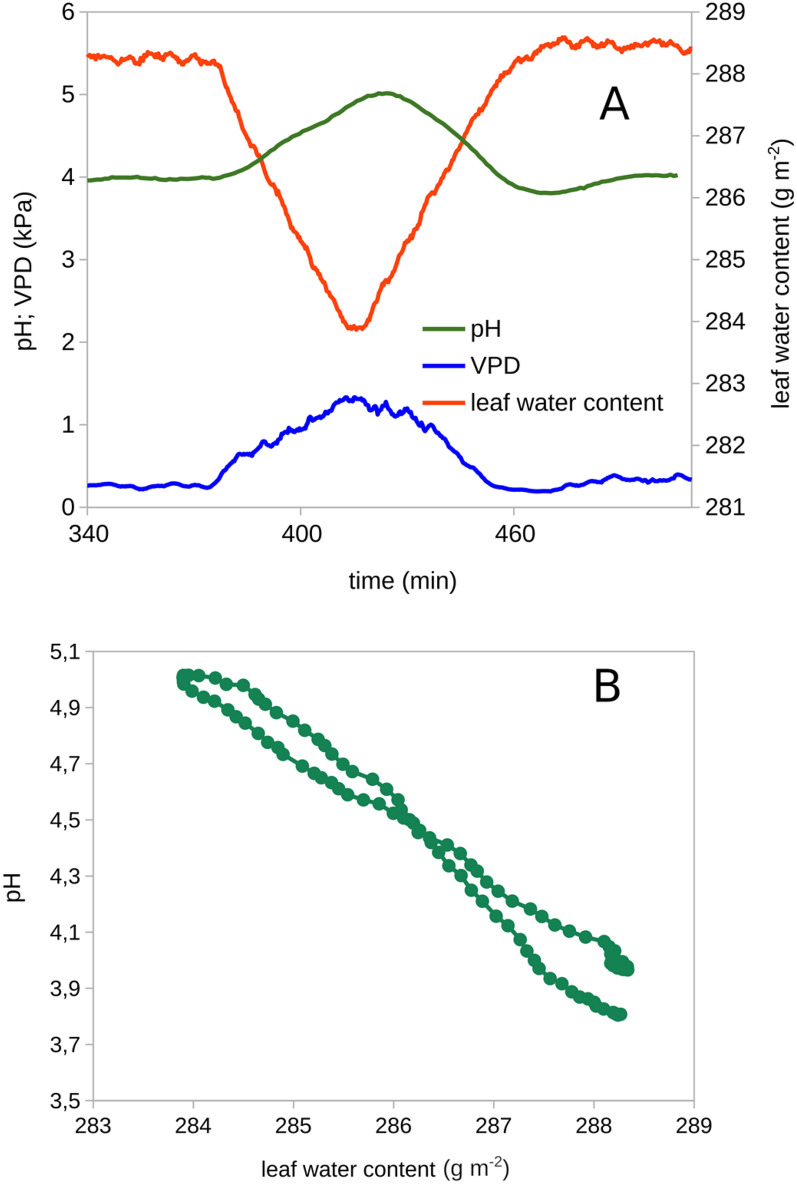
**A** Recording of apoplastic pH of the lower leaf epidermis by fluorescence ratio imaging using OregonGreen under feedback-controlled leaf water content (LWC). LWC set-point was varied by a programmed ramp from 100% full saturation under low VPD to 98.5% saturation within 40 min. and in reverse to achieve constant rates of change of LWC. **B** Relation between LWC and apopastic pH. Same data as panel **A**. Here the the dose response relation observed during slowly changing LWC is visualized

More complex experimental manipulations of LWC are also possible. Programmed ramps of LWC were executed to slowly change LWC (Fig. 4A). This experiment was designed to assess the effect of reductions in LWC on apoplastic pH. It is well established that pH_apo_ responds to salinity, reductions osmotic stress and high VPD with substantial transient alkalization [4, 6, 7]. These stresses however are comprised of sub-stresses (e.g. ionic, osmotic, dehydration) with different kinetics and amplitudes which are intransparent to the experimenter, hindering a clear causal assignment of the observed pH responses. The idea behind this experiment was to assess the pH_apo_ response to an experimentally isolated and repeatably controlled reduction in LWC. By slowly changing LWC allowing enough time for equilibration, it was attempted to derive a dose–response relationship between LWC and pH_apo_ (Fig. 4B). This experiment demonstrates firstly that more complex and methodologically useful transitions of LWC can be experimentally imposed. Additionally, the apparent co-variation of LWC and pH_apo_ demonstrates the usefulness of this non-invasive control technique to study functional relationships between delicate and easily disturbed properties in combination with other non-invasive measurements. Several possible method combinations appear feasible like the use of genetically encoded fluorescent sensors for apoplastic or symplastic ion concentrations or electrophysiological methods using microelectrodes and of course many cuvette based gas exchange measurements and chlorophyll fluorescence probing of photosynthetic energy conversion.

## Conclusions

Bringing LWC under experimental control helps to better assess its role as an input into signaling and regulatory processes. The conventional approach of varying LWC by changing external environmental variables, like VPD, suffers from the unpredictable physiological feedback of the research object. Stomatal responses and other homeostatic feedback responses will defy attempts to precisely and repeatably control LWC. The proposed method in contrast allows to truly treat LWC as an experimental factor to assess its effect on biological responses (e.g. stomatal movements and osmotic adjustment) and putative intermediate signaling events like changes in [ABA] and pH_apo_.

## Data Availability

All data generated or analysed during this study are included in this published article in graphical form. The numerical datasets generated during and/or analysed during the current study are available from the corresponding author on reasonable request.
